# Cross-Species Behavioral Analysis via Multidimensional Knowledge Transfer

**DOI:** 10.1109/mis.2026.3692032

**Published:** 2026-05-12

**Authors:** Yang Liu, Luke Shaw, Haotian Liu, Kuan Hong Wang, Feng Vankee-Lin, Guoying Zhao

**Affiliations:** University of Oulu, 90570, Oulu, Finland, and Stanford University, Palo Alto, CA, 94304, USA; University of Rochester Medical Center, Rochester, NY, 14642, USA; University of Oulu, 90570, Oulu, Finland; University of Rochester Medical Center, Rochester, NY, 14642, USA; Stanford University, Palo Alto, CA, 94304, USA, and VA Palo Alto, Palo Alto, CA, 94304, USA; ELLIS Institute Finland, 02150, Espoo, Finland, and University of Oulu, 90570, Oulu, Finland

## Abstract

Computer vision tools can provide objective, reproducible, and scalable measurements for understanding animal behaviors, which is a fundamental challenge in cross-species research. Existing approaches primarily emphasize body detection and tracking, with limited advancement in integrative behavioral pattern analysis due to sparse annotations and substantial variability in species’ physical and cognitive traits. In this article, we conduct two pilot studies for cross-species behavioral analysis within primates using multidimensional transfer learning. First, prior knowledge of physiological similarities between humans and monkeys is introduced to adjust the representation learning for macaque facial expression classification under limited labeled data. Second, we improve our approach by leveraging the foundation model and self-supervised strategy to mitigate appearance variations across primate species for downstream marmoset engagement level estimation. Experiments on public and private cross-primates datasets demonstrate the effectiveness of our framework, highlighting the potential of knowledge transfer to bridge the gap between species-specific behavioral analysis and generalizable models.

Understanding cross-species behaviors, such as emotional expressions and cognitive actions, is a critical endeavor that can not only help us study ecology and neuroscience but also discover shared mechanisms to promote animal welfare and human health.^1^ While significant progress has been made in detecting and tracking behaviors through computer vision approaches within individual species,^2^ the ability to generalize these insights for analyzing cross-species behaviors remains a significant challenge. In this work, we focus on cross-species behavioral transfer within primates, including humans, macaques, and marmosets, where partially homologous facial expression coding systems provide biologically grounded anchors for representation transfer.

## RELATED CONTEXT

Prior work in animal behavior analysis has largely focused on detection, tracking, or pose estimation within individual species, with limited emphasis on transferable behavioral representations across species.^3,4,5^ Recent advances in cross-species perception and foundation models have demonstrated the potential of large-scale pretraining to improve generalization,^6,7^ yet these approaches typically lack explicit mechanisms for incorporating structured behavioral priors or for fine-grained knowledge transfer. A more comprehensive discussion of cross-species behavioral analyses, including demonstrations involving nonprimate species and alternative expression components, can be found in Liu et al.^1^

Despite recent progress, one of the primary challenges is the scarcity of high-quality, annotated datasets for different animal species.^3^ Unlike human behavioral datasets, such as facial expressions and body gestures, which are often abundant and well-labeled,^8^ animal datasets are typically limited in size, diversity, and granularity. This shortage is due to the difficulties associated with data collection in natural or controlled environments, including the need for specialized equipment, ethical considerations, and the labor-intensive process of manual annotation. Although there have been a few large-scale animal datasets, like *Animal Kingdom*,^4^ that involve annotated faces or behaviors of various species, the vast diversity in physical, cognitive, and emotional traits among them complicates the development of robust data-driven models to capture the full spectrum of cross-species behaviors. Moreover, existing approaches for human behavioral analysis predominantly follow a supervised manner and often fail to adapt to other species because of unique characteristics and inconsistent label types across different species.^9^ While recent self-supervised learning methods have been explored to offer a promising solution by leveraging unlabeled data, they either perform basic tasks, like cross-species pose estimation, or only focus on one specific species without considering shared behavioral patterns across species,^5^ limiting the effectiveness of pretraining and the generalization capability in complex downstream tasks.

Another significant challenge in cross-species behavioral analysis is the inherent variability in facial/body appearance and movements across different species. These variations arise from differences in anatomy, physiology, cognitive abilities, and environmental contexts, creating substantial domain differences that limit the effectiveness of traditional transfer learning techniques.^10^ For instance, a model trained on human facial expressions may struggle to generalize to other species due to differences in facial musculature and behavioral semantics. This domain gap is further exacerbated by the lack of shared prior knowledge that can guide the learning process. Introducing domain-specific prior knowledge, such as the Facial Action Coding System (FACS) across species,^11^ can help bridge this gap by providing a structured framework for understanding and interpreting behaviors. However, how to link the shared knowledge of cross-species FACS and integrate it into the transfer learning model remains underexplored. Lately, foundation models, which are pretrained on large-scale datasets and fine-tuned for specific tasks, offer a promising alternative to address domain differences.^6^ By leveraging vast amounts of unlabeled data, foundation models can learn generalizable representations that capture environmental and ecological attributes across species.^7^ These representations can then be adapted to downstream tasks by learning shared behavioral patterns with minimal labeled data, reducing the reliance on species-specific prior knowledge.

In this article, we propose a multidimensional transfer learning framework to enhance cross-species behavioral analysis. The proposed framework integrates concept-level priors, parameter-level adaptation, foundation models, and self-supervised learning into a unified system, providing a generalizable artificial intelligence (AI) strategy for domains where labels are limited, semantics vary across domains, and interpretability remains critical. We focus on primate facial expressions because they provide a well-characterized and anatomically grounded expression component with established cross-species correspondence (e.g., MaqFACS^11^ and CalliFACS^12^). The main contributions are as follows:

- We integrate structured concept-level prior knowledge with parameter-level transfer to improve representation learning under domain shift and label scarcity.
- The use of foundation models and self-supervised learning is explored to address appearance variations across species, which effectively use limited labeled data for downstream behavioral understanding tasks.
- Experiments of macaque facial expression classification and marmoset engagement estimation on two datasets demonstrate the effectiveness of the proposed methods, illustrating a promising direction for scalable and generalizable cross-species behavioral understanding.

## PROPOSED METHOD

### Framework Overview

To address the above-mentioned challenges, we propose a multidimensional transfer learning framework that systematically integrates concept-level and parameter-level knowledge transfer. At the concept level, we leverage standardized alignment of behavioral semantics to identify conserved expressive patterns across species. The parameter level progressively specializes model representations from generic visual features to species-specific behavioral traits, optimizing both in generalization and specificity. The framework provides a robust and flexible pipeline, enabling effective knowledge transfer of universal behavioral principles while adapting to species-specific dynamics. The current implementation focuses on primate species, where facial expression correspondences provide biologically interpretable anchors for cross-species transfer.

**FIGURE 1.**
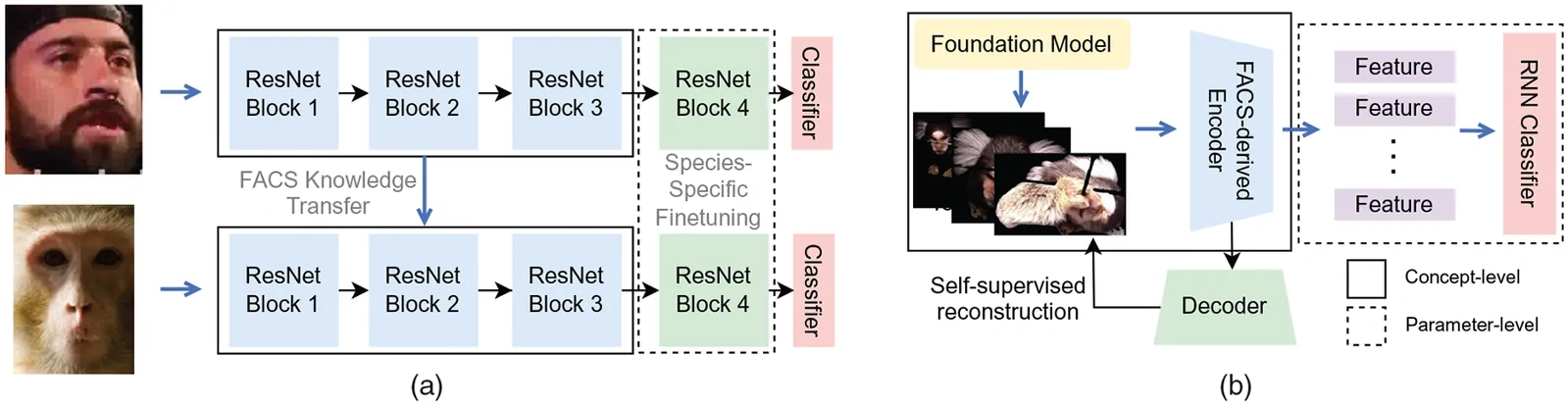
Multidimensional knowledge transfer. (a) FACS-informed cross-species representation learning. (b) Semantic-aware species-specific adaptation. NOTE: FACS transfer represents one example of a concept-level anchor; other expression components (for example, body movements or locomotion dynamics) may be substituted for nonprimate species.

Specifically, the proposed framework follows a staged transfer-learning process across three domains, as shown in Figure 1. First, a human facial-expression model trained on large-scale affective datasets provides general facial representation priors. Second, cross-species knowledge distillation transfers these representations to macaque facial expression recognition using FACS-informed semantic correspondence. This step adapts the model toward primate-specific facial morphology while preserving general facial dynamics. Finally, the learned representation is further adapted to marmoset engagement estimation through a semantic-aware adaptation stage that integrates spatial priors from a visual–language foundation model and temporal modeling. This hierarchical transfer strategy progressively reduces domain gaps from humans to macaques and then to marmosets.

In this work, “multidimensional transfer” specifically refers to the integration of: 1) concept-level semantic alignment via FACS, 2) parameter-level adaptation through knowledge distillation, 3) foundation model-based spatial priors, and 4) self-supervised temporal representation learning. These components are designed to be modular and complementary, enabling flexible adaptation across species.

### FACS-Informed Cross-Species Representation Learning

The FACS provides a standardized, anatomically grounded method for analyzing facial expressions by decomposing them into fundamental muscle movements, or action units (AUs). This systematic approach enables precise and objective comparisons across species, making it particularly valuable for cross-species behavioral research. In macaques, the MaqFACS^11^ maps facial movements to homologous muscle activations, aligning with human FACS where applicable. For instance, shared AUs, such as brow raising (AU1 + 2) or lip parting (AU25), highlight conserved mechanisms in emotional signaling across primates.^11^ By applying shared AU definitions, we establish a concept-level alignment between human and macaque facial expressions to exploit prior knowledge of human facial models while accounting for species-specific variations, ensuring robust representation learning even with limited macaque-labeled data.

#### Preprocessing Validation

**FIGURE 2.**
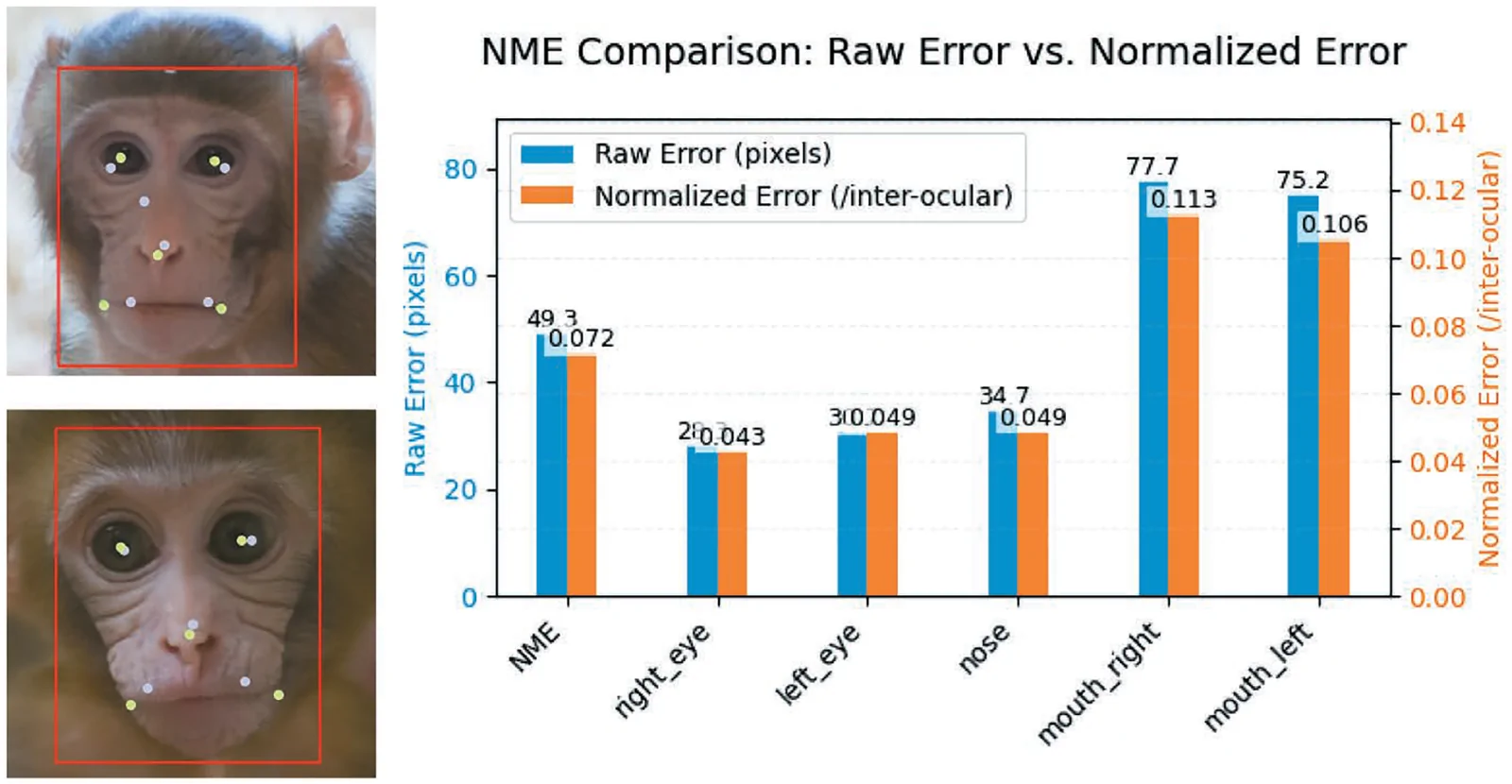
Performance of human-based RetinaFace for macaque face detection and facial landmark localization. Blue points denote model’s prediction, green points denote manual ground truth. NME is defined as the average Euclidean distance between predicted and ground-truth landmark locations across all landmarks, normalized by the inter-ocular distance.

Similar to human studies, obtaining facial regions is the first important step for macaque facial analysis. Since there is no well-trained monkey face detector, we validate the performance of the pretrained human face detection model on macaque facial images without fine-tuning. In this case, we apply the *RetinaFace*^13^ as our base model. The validation process consists of two key stages. First, we manually annotate bounding boxes and five facial landmarks (including left/right eye centers, nose tip, left/right mouth corners) for each image in the dataset, using the Microsoft Visual Object Tagging Tool and Computer Vision Annotation Tool, respectively. Second, the performance of face detection (i.e., four-pixel coordinate) and landmark localization is evaluated using the normalized mean error (NME) metric, defined as the average Euclidean distance between predicted and ground-truth landmarks, normalized by the interocular distance. As shown in Figure 2, the RetinaFace achieves reasonable zero-shot performance on macaque faces, confirming moderate generalization to macaque faces despite domain gaps.

#### Pair-Wise Knowledge Distillation

Ideally, with the cross-species FACS as shared semantic anchors, we can establish the knowledge transfer between humans and macaques by aligning embedding spaces, pulling together same-AU pairs across species in a contrastive learning manner. However, the incomplete consistency of FACS caused by species differences and the lack of AU-annotated macaque datasets limits this approach. Alternatively, inspired by Zhang et al.,^14^ we designed a pair-wise knowledge distillation module that transfers knowledge from a pretrained human facial expression model to a macaque facial expression model by leveraging the anatomical similarities between human and macaque faces. The process involves a dual-branch training scheme where pairs of human and macaque faces are input simultaneously.

Let *f*_*h*_(·;*θ*_*h*_) and *f*_*m*_(·;*θ*_*m*_) denote the human and macaque branches implemented as ResNet-50, where *θ*_*h*_ is fixed and *θ*_*m*_ is partially frozen

(1) $$\theta_{m}=\theta_{m}^{\text{frozen}},\theta_{m}^{\text{trainable}}.$$

The knowledge transfer is achieved by minimizing the Kullback–Leibler (KL) divergence between the softened output probabilities of the two branches defined as

(2) $${ℒ}_{\text{KD}}=\lambda T^{2}\cdot D_{\text{KL}}\left(p_{h}^{T}|p_{m}^{T}\right)+(1-\lambda ){ℒ}_{\text{CE}}\left(y_{m},{\overset{ˆ}{y}}_{m}\right)$$

where $p_{h}^{T}=\text{softmax}{\left(z_{h}/T\right)}_{,}p_{m}^{T}=\text{softmax}\left(z_{m}/T\right)$ are temperature-scaled probabilities, *D*_KL_ computes KL divergence, ${ℒ}_{\text{CE}}$ is standard cross-entropy loss, *T* is the temperature hyperparameter, and λ∈[0,1] balances distillation and classification. An optional L2 loss aligns intermediate features

(3) $${ℒ}_{\text{feat}}=\frac{1}{N}\sum i=1^{N}{\left|\phi_{h}\left(x_{h}^{\left(i\right)}\right)-\phi_{m}\left(x_{m}^{\left(i\right)}\right)\right|}_{2}^{2}$$

where *θ*(·) donotes feature maps from a middle layer. The complete optimization target is formulated as

(4) $${ℒ}_{\text{FACS}}={ℒ}_{\text{KD}}+\alpha {ℒ}_{\text{feat}}.$$

This module enables efficient FACS-informed cross-species knowledge transfer while preserving species-specific characteristics through partial fine-tuning.

### Semantic-Aware Species-Specific Adaptation

#### Role of Macaque Data

Building upon human–macaque knowledge transfer, we extend our multidimensional framework to marmoset engagement estimation. While MaqFACS bridges humans and macaques through homologous AUs, marmosets lack direct anatomical counterparts for many primate AUs. The existing marmoset FACS, i.e., Calli-FACS,^12^ identifies species-specific gesture movements (e.g., ear flattening or whisker twitch) that convey emotional states but diverge from primate AU semantics. Additionally, dynamic engagement behaviors require further temporal modeling of marmosets. Therefore, macaque facial expression data are not used to directly supervise or label marmoset engagement states. Instead, macaque data serve as an intermediate domain that regularizes representation learning toward primate-specific appearance and motion statistics before adaptation to marmoset behaviors. This intermediate transfer helps reduce the domain gap between human-centric visual features and marmoset-specific engagement cues. As a result, the learned representations become more robust under limited marmoset annotations without assuming semantic equivalence between macaque facial expressions and marmoset engagement states. Conceptually, this stage functions as a coarse-to-fine transfer process, where human facial priors are first adapted to primate morphology through macaque data and then further specialized for marmoset data. To this end, we proposed a semantic-aware adaptation strategy that combines foundation models and self-supervised learning.

#### Foundation Model Integration

We employ a visual–language foundation model (Grounded SAM^15^) to provide coarse spatial priors for marmoset behavior analysis via prompt-based segmentation (e.g., [monkey] + [marshmallow]/[cricket]). Importantly, Grounded SAM is treated as a frozen and task-agnostic module, and is not optimized jointly with the downstream model. The segmentation masks are used solely to provide spatial focus for feature extraction, rather than serving as supervisory signals. To reduce potential dependency on segmentation quality, the downstream learning is explicitly decoupled from the correctness of the mask. The temporal modeling and self-supervised reconstruction operate on masked visual features rather than directly on segmentation outputs, ensuring that moderate segmentation errors introduce noise but do not propagate semantic bias. Furthermore, the proposed framework is modular, and the spatial prior component can be replaced by alternative region proposals (e.g., bounding boxes or heuristic cropping) without altering the core transfer learning mechanism.

For each frame *x*_*t*_, the model generates a segmentation mask *M*_*t*_

(5) $$M_{t}=\text{LangSAM}\left(x_{t},p\right).$$

The masked features are extracted via

(6) $$f_{t}^{\text{masked}}=\text{ResNet}\left(x_{t}⊙M_{t}\right).$$

Importantly, the segmentation mask is not treated as a supervisory signal. Moderate segmentation errors may introduce noise in feature extraction but do not directly propagate semantic bias into engagement predictions, as the downstream GRU-based temporal model and self-supervised reconstruction objective operate on masked visual features rather than segmentation correctness. This design ensures robustness and generalization, particularly under limited and heterogeneous animal data, in our case. This self-supervised objective is applied to both MarmoCatch clips^16^ and auxiliary marmoset frames from the DeepLabCut benchmark dataset,^17^ without using pose or identity annotations.

#### Self-Supervised Motion Dynamics

To compensate the limited marmoset data, a reconstruction task learns motion features as follows:

(7) $${ℒ}_{\text{recon}}=\sum_{t=1}^{T}{\left|\text{Dec}\left(ft^{\text{masked}}\right)-x_{t+1}\right|}_{1}$$

where Dec(·) is a convolutional decoder.

#### Temporal Modeling

A GRU network is exploited to predict engagement scores *s* from sequential features

(8) $$s=\sigma \left(\text{GRU}\left(f_{1}^{\text{masked}},\ldots ,f_{k}^{\text{masked}}\right)\cdot w\right)$$

where *k* = 16 is the length of sampled video frames in our case. The total loss combines reconstruction and classification

(9) $${ℒ}_{\text{ADPT}}=\gamma {ℒ}_{\text{recon}}+{ℒ}_{\text{CE}}.$$

This strategy bypasses explicit cross-species representation alignment, instead leveraging foundation models and self-supervision to adapt to marmoset-specific behaviors, highlighting the flexibility of the multidimensional knowledge transfer.

##### Training Strategy

The training objectives correspond to different stages of the proposed transfer-learning pipeline. During the human-to-macaque adaptation stage, the model is optimized using the FACS-informed transfer objective ${ℒ}_{\text{FACS}}$ which combines knowledge distillation and feature alignment to transfer facial representation priors while preserving species-specific characteristics. In the downstream marmoset engagement estimation stage, the model is trained using the adaptation objective ${ℒ}_{\text{ADPT}}$, which integrates a self-supervised reconstruction loss and a supervised engagement classification loss. This staged optimization strategy enables the model to progressively reduce domain gaps across species while maintaining stable representation learning under limited labeled data. In this staged optimization process, macaque data function as an intermediate alignment domain that regularizes representations before the marmoset engagement estimation stage.

## EXPERIMENT

### Datasets

To evaluate the performance of our cross-species knowledge transfer framework, we implemented experiments using three animal behavior datasets, i.e., MacaqueFace, MarmoCatch,^16^ and DeepLabCut Marmoset.^17^

*MacaqueFace* is a public macaque dataset, including 3896 images of 81 individual rhesus macaques from colony A and colony B (threat neutral faces pairs without labels), accompanying metadata of identity, sex, age, and colony affiliation, as well as behavioral annotations of facial expression, head orientation, and gaze direction. In our experiments, 3034 images from 52 individuals and 836 images from 21 individuals in colonies A were used for training and testing, respectively, evaluating performance on eight-class classification (i.e., *neutral*, *suckling*, *expectation*, *eating*, *tongue*, *yawn*, *threat*, *lipsmack*).

*MarmoCatch* is a private marmoset dataset, involving food retrieval behaviors for marshmallows (72 video clips) or crickets (48 video clips). It consists of 120 videos from three trial groups in four different view angles, plus annotations of retrieval success, prereach focus (engagement), reach ease, and retrieval ease. In our experiments, 96 and 24 clips were used for training and testing, respectively, evaluating performance on three-class (i.e., *distracted*, *semiengaged*, and *engaged*) engagement level classification.

*DeepLabCut Marmoset* is part of the public cross-species dataset, i.e., DeepLabCut Benchmark,^17^ for animal behavioral analysis. These data were collected from common marmosets (*Callithrix jacchus*) recorded in laboratory settings using Kinect V2 cameras at 1080p resolution and 30-Hz frame rate, across three different colonies. While the original dataset includes manually annotated body landmarks for pose estimation, we did not use any pose labels or behavioral annotations in this study. Instead, the raw video frames were treated as unlabeled inputs and are used exclusively for self-supervised reconstruction and feature regularization, without contributing to supervised engagement loss or evaluation.

### Implementation Details

The proposed methods were implemented with the Pytorch platform and trained using one Nvidia A100 GPU. For macaque facial expression classification, we fine-tuned a ResNet-50 backbone (pretrained on Aff-Wild2) with the first three convolutional blocks frozen, using stochastic gradient descent optimizer with momentum as 0.9, initial learning rate as 0.001 decaying to 1*e* − 4, *λ* as set to 0.7, and *T* as 3 on RetinaFace detected 224 × 224 facial images, batch size is set to 256.

The marmoset engagement estimation task employs language-supported Grounded SAM for prompt-based segmentation of 16 uniformly sampled frames per clip, processed by a ResNet-50 feature extractor and GRU with 128 hidden units, and trained with Adam optimizer using a learning rate of 5*e* − 4, and *γ* set to 0.5 for three-class engagement prediction. Frames from the DeepLabCut Marmoset were excluded from supervised loss computation and evaluation, and were processed using the same frame sampling and preprocessing pipeline as MarmoCatch.

We included the recurrent video transformer (RVT)^2^ as a representative baseline for estimating spatiotemporal engagement. The RVT model was initialized from human-centric pretraining and further fine-tuned on marmoset engagement data without incorporating the proposed multidimensional transfer learning framework. Therefore, the comparison evaluates whether the proposed structured transfer learning framework provides additional benefits beyond conventional pretraining and fine-tuning.

### Ablation Study

To validate the effectiveness of the proposed components in our framework, we conducted ablation studies on both the macaque facial expression classification and marmoset engagement estimation tasks. As shown in Table 1, the FACS pretraining with human AU knowledge boosts performance, and the feature alignment provides intermediate matching, showing cross-species anatomical alignment is effective. Freezing early layers preserves general features while accounting for species-specific variations.

To further evaluate the reliance on Grounded SAM, we conducted additional ablation experiments by introducing controlled perturbations to the segmentation masks. Table 2 evaluates the impact of segmentation masks and additional cross-species training data on marmoset engagement estimation. Adding semantic masks significantly improves performance, demonstrating that spatial localization provided by foundation models enhances feature extraction.

Building on this, to assess robustness to segmentation quality, we simulated degradation using random spatial mask dropout, where each pixel within the segmentation mask is independently removed with a probability (*P* = 0.3), mimicking partial detection failures. The performance only degrades marginally compared to the original mask setting, indicating that the proposed framework does not rely on precise segmentation boundaries, but instead benefits from coarse spatial consistency. This suggests that Grounded SAM functions as a weak spatial prior rather than a critical dependency.

Additionally, we find that incorporating additional unlabeled macaque and marmoset data further improves both accuracy and F1 score, validating the effectiveness of cross-species transfer and self-supervised learning in mitigating data scarcity. Notably, the performance gain from incorporating macaque data reflects improved representation robustness rather than semantic transfer, as no macaque engagement labels are used in marmoset training.

### Evaluation of Macaque Facial Expression Recognition

**FIGURE 3.**
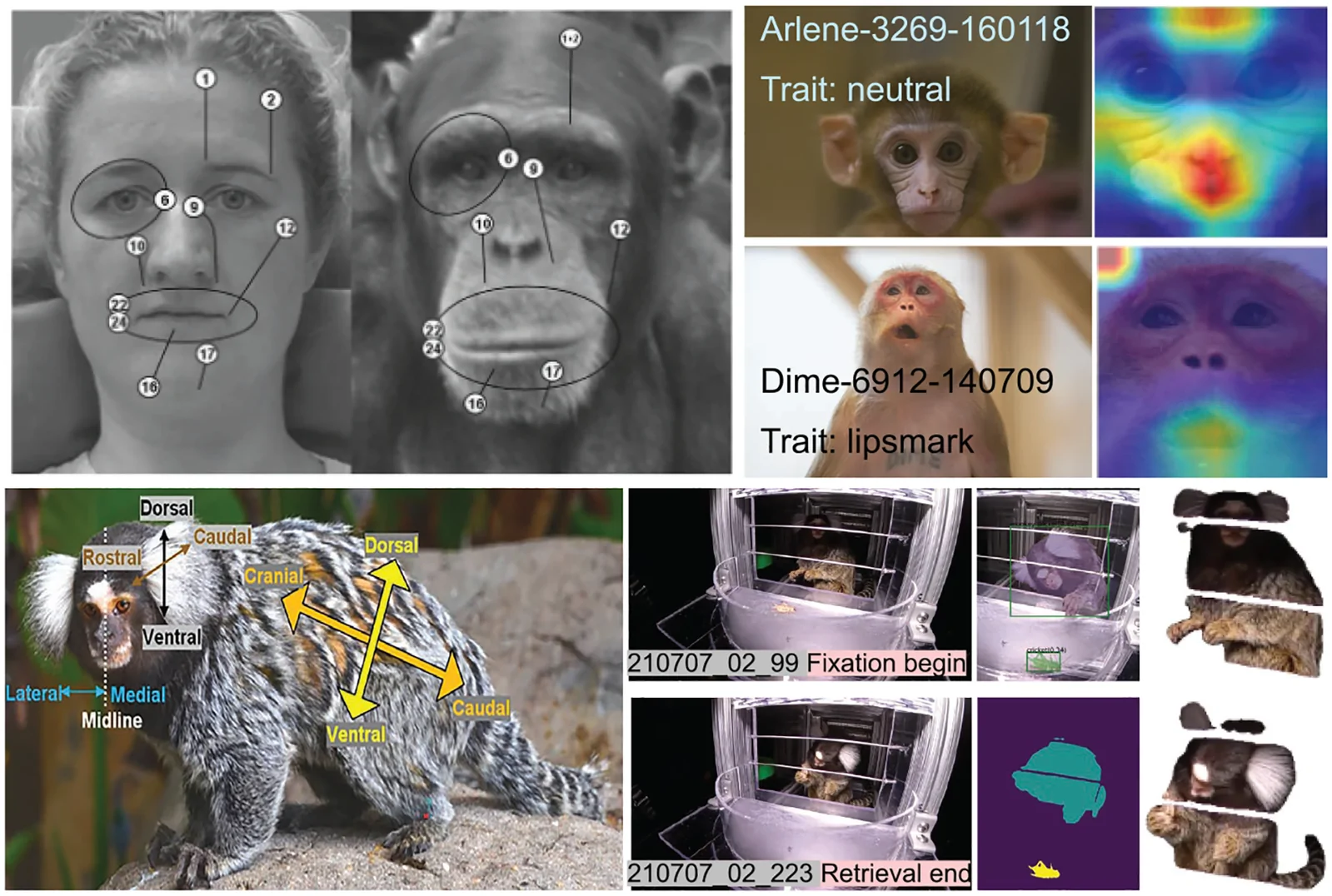
(Left top) Example of FACS versus MaqFace. (Right top) Feature visualization. (Bottom) Example of CalliFACS and semantic mask visualization. Segmentation masks are used as spatial priors for feature extraction rather than as supervision signals, and engagement prediction is learned independently through temporal modeling.

As presented in Table 3, the proposed method outperforms the baseline and state-of-the-art approaches, including vanilla ResNet-50, MTAC^18^ and HSEmotion,^19^ highlighting the generalization of our FACS-informed knowledge transfer (concept-level) and pair-wise knowledge distillation (parameter-level). The visualization in Figure 3 shows that the model can effectively represent macaque facial expressions through our cross-species FACS-informed knowledge transfer.

### Evaluation of Marmoset Engagement Estimation

Table 4 shows that the proposed method achieves over 60% accuracy, surpassing the baseline 3-D-convolutional neural network (CNN) and human engagement estimation approach RVT.^2^ RVT is selected as a baseline because it represents a strong, widely adopted spatiotemporal transformer architecture for engagement-related video understanding. Using RVT allows us to assess whether performance gains arise from architectural scale alone or from the proposed multidimensional transfer strategy. The foundation model’s prompt-based segmentation enables robust feature extraction from limited species-specific data, as shown in Figure 3. The integration of temporal modeling captures dynamic engagement behaviors, further improving the performance. We clarify that RVT already benefits from standard transfer learning (human pretraining followed by fine-tuning on marmoset data). The RVT performance reflects direct supervised learning on limited marmoset labels without intermediate cross-species alignment, highlighting the impact of the proposed transfer framework rather than backbone capacity. In contrast, our method introduces a structured multidimensional transfer framework. The observed performance gain thus reflects the advantage of explicitly modeling cross-species knowledge transfer rather than relying solely on conventional pretraining and fine-tuning. Moreover, we conducted experiments regarding different targets, as shown in Table 5. The engagement prediction varies slightly by marshmallow and cricket, which demonstrates our model’s generalization capabilities. The result of binary target differentiation reveals the potential of our method in performing complex downstream tasks.

### Discussion

The current study conducts concept-level transfer using FACS-informed facial representations, which naturally constrains semantic alignment to primates with partially homologous facial musculature. This limitation, however, relates to the selected expression component rather than to the proposed transfer-learning paradigm. As elaborated in our recent perspective on multidimensional transfer learning for cross-species behavioral research,^1^ nonprimate demonstrations using alternative expression components, such as locomotion trajectories and body-movement dynamics, have shown that the same framework can support cross-species modeling across rodents, insects, and humans by replacing facial semantics with species-appropriate behavioral elements. These demonstrations highlight that the framework is feasible to other interpretable anchors for achieving cross-species reliability and translational validity.

From a generalization perspective, the visual–language foundation model serves a complementary role to concept-level transfer. Rather than encoding behavioral semantics, it provides a reusable spatial abstraction that can be replaced by alternative detectors or segmentation tools. This aligns with recent frameworks that treat foundation models as modular utilities rather than task-specific predictors, allowing the core behavioral inference model to remain species-adaptive even when upstream components vary. Importantly, our ablation results further confirm that the proposed framework does not rely on precise segmentation quality, as performance remains stable under degraded or perturbed masks. This suggests that the foundation model functions as a coarse spatial prior rather than a critical dependency. Conceptually, the macaque dataset serves as an intermediate alignment stage in a coarse-to-fine transfer process, facilitating adaptation from human-centric priors to marmoset-specific behaviors without requiring direct cross-species semantic correspondence. While the present study focuses on primates due to the availability of homologous facial expression annotations, the proposed framework is, in principle, extendable to other species by redefining appropriate behavioral anchors (e.g., locomotion^20^).

## CONCLUSION

In this work, we proposed a multidimensional transfer learning framework to address challenges in cross-species behavioral analysis, including data scarcity and appearance variations. By integrating FACS-informed prior knowledge and leveraging foundation models with self-supervised learning, our approach achieved state-of-the-art performance in macaque facial expression classification and marmoset engagement estimation, highlighting the potential of knowledge transfer to bridge species-specific gaps and enable scalable behavioral understanding. Future work will explore extending this framework to additional species and more complex behavioral tasks. More broadly, the proposed framework provides a transferable strategy for learning across heterogeneous domains with limited supervision, extending beyond ecological settings. Future work will further investigate alternative spatial priors and stronger baseline adaptations to systematically evaluate the contribution of each module in more diverse cross-species settings.

## Figures and Tables

**TABLE 1.** Component contribution of FACS-informed representation.

Configuration	F1-Score	Accuracy (%)
Without FACS pretraining	0.591	62.1
With FACS pretraining	0.656	68.3
Without block freezing	0.614	64.2
With block freezing	0.638	66.7
Without feature alignment	0.634	66.2
With feature alignment	0.692	71.8

**TABLE 2.** Component contribution of semantic-aware adaptation.

Configuration	F1-Score	Accuracy (%)
Without segmentation masks	0.497	51.8
With mask dropout	0.512	53.4
With segmentation masks	0.521	53.9
Without cross-species data	0.546	55.2
With cross-species data	0.551	56.4

**TABLE 3.** Comparison on macaque facial expression classification.

Method	F1-Score	Accuracy (%)
ResNet-50	0.616	64.5
MTAC	0.685	71.8
HSEmotion	0.677	70.1
Ours	0.715	74.5

**TABLE 4.** Comparison on marmoset engagement estimation.

Method	F1-Score	Accuracy (%)
3 D-CNN	0.410	42.2
RVT	0.502	51.3
Ours	0.594	60.9

**TABLE 5.** Impact and binary differentiation of targets.

Target	F1-Score	Accuracy (%)
Marshmallow	0.606	61.5
Cricket	0.588	60.2
Binary target differentiation	0.667	68.5
